# Charting the Future of Community Health Promotion: Recommendations From the National Expert Panel on Community Health Promotion

**Published:** 2007-06-15

**Authors:** Leandris Liburd, Janet L Collins, H. Wayne Giles, Karen P Voetsch, Amanda M Navarro

**Affiliations:** Division of Adult and Community Health, National Center for Chronic Disease Prevention and Health Promotion, Centers for Disease Control and Prevention; Coordinating Center for Health Promotion, National Center for Chronic Disease Prevention and Health Promotion, Centers for Disease Control and Prevention, Atlanta, Ga; Division of Adult and Community Health, National Center for Chronic Disease Prevention and Health Promotion, Centers for Disease Control and Prevention, Atlanta, Ga; Division of Adult and Community Health, National Center for Chronic Disease Prevention and Health Promotion, Centers for Disease Control and Prevention, Atlanta, Ga; Public Health Division, Northrop Grumman Corporation, Atlanta, Ga

## Abstract

In the decades since chronic illnesses replaced infectious diseases as the leading causes of death, public health researchers, particularly those in the field of health promotion and chronic disease prevention, have shifted their focus from the individual to the community in recognition that community-level changes will foster and sustain individual behavior change. The former emphasis on individual lifestyle change has been broadened to include social and environmental factors, often without increased resources. To find new ways to support community health promotion at the national level, the National Center for Chronic Disease Prevention and Health Promotion and the Division of Adult and Community Health invited an external panel of experts to participate in the National Expert Panel on Community Health Promotion. This article highlights the process through which the expert panel developed its eight recommendations. The recommendations include issues related to community-based participatory research and surveillance, training and capacity building, new approaches for health and wellness, and changes in federal investments. They illustrate the steps needed to broaden the traditional scope of public health and to advance a new vision for improving community health and wellness.

## Background

The shift from infectious diseases to chronic illnesses as the leading causes of death required a new strategic direction for the field of public health (1). Identifying a single agent or threat was no longer sufficient to address the complex factors underlying the rise in chronic disease burden and deaths (1). This reality, coupled with a greater understanding of the link between behaviors and chronic disease and an expanded appreciation for the contributions of the social sciences, led to the growth of health promotion in public health science and practice (2). Health promotion has its roots in an ecological framework; that is, it emphasizes the interaction between the individual and the physical and social environment and addresses these determinants of health through social networks, agencies and institutions, neighborhoods, and communities. Recognizing the interaction of these environmental factors offers the possibility of new approaches to influencing and supporting individuals, as members of communities, to adopt and maintain healthy lifestyles (3).

Community health promotion emphasizes the social, cultural, and environmental contexts that shape health status and works through collaborative partnerships to improve the health of a population within a defined geographic area (3). Community-based participatory strategies, such as community organization and mobilization, bring to bear shared values and experiences of community members, who are viewed as having the best knowledge of and perspective on improving the health of their community (4). The community is regarded as an influential determinant of health, and community members are involved in all aspects of public health research, interventions, and evaluations (4).

Health promotion's foundation rests in several landmark documents, including the Lalonde report from Canada, the Ottawa Charter, the Canadian Epp Framework, and *Healthy People: The Surgeon General's Report on Health Promotion and Disease Prevention* (5-8). Each called for new public health strategies that focus not only on personal behavior change but also on the social, psychological, and economic determinants of health (9). As a response to this movement, in 1981, the Centers for Disease Control and Prevention (CDC) established a center for health promotion and education that focused on health issues such as sexual and contraceptive behavior, nutrition, smoking, alcohol use, physical fitness, stress, violence, injury, and other risk factors of public health significance. This center provided the only source of federal funds to state agencies to support health education and health promotion through the Health Education and Risk Reduction grant program.

In subsequent years, the center expanded its scope to support community-wide planning (with the creation of the Planned Approach to Community Health [PATCH] model) and state-based public health surveillance (with the creation of the Behavioral Risk Factor Surveillance System and other key surveillance programs) (10). By 1988, the center, by then known as the National Center for Chronic Disease Prevention and Health Promotion (NCCDPHP), became increasingly disease- and risk factor-specific, with its existing health education and community health promotion expertise spread across its categorical programs. Although this expertise has become integral to prevention research and categorical health programs throughout the center, a point of access for public health scientists and practitioners seeking to improve the efficacy and effectiveness of community health promotion that was once clearly concentrated is now diffuse and dispersed.

As prevalence rates and costs of chronic diseases continue to rise, societal institutions — such as businesses, health care settings, and schools — increasingly demand that public health programs show health impact and cost savings in the short term. The challenge for NCCDPHP and other leaders in public health is to emphasize the important role of long-term community health promotion in addressing the social and environmental determinants of health in an atmosphere that demands evidence of health impact and return on investment. The Institute of Medicine's report *The Future of the Public's Health in the 21st Century* and several recent articles challenge the public health community to establish a new vision for public health science and practice that measures and addresses these social and environmental determinants and supports the role of the community in improving health and wellness (10-13). NCCDPHP and its Division of Adult and Community Health (DACH) responded to this challenge by inviting a panel of experts to act as an external review board and to provide guidance to advance community health promotion (14). The expert panel was charged with the following: 1) identifying and summarizing gaps in the field of community health promotion, as well as relevant and cutting-edge initiatives for community health promotion, and 2) developing and prioritizing recommendations for center-wide activities to promote community health within the next 3 to 5 years.

## Methods

In March 2006, 25 people representing various health care sectors and broad areas of public health and community expertise participated in the National Expert Panel on Community Health Promotion. Those invited to the 2-day meeting included experts on community-based participatory research, local community-based practice, aging, and mental health; leaders from community-based and nonprofit organizations; and state and local health department representatives. The participants were selected on the basis of their current affiliation, a specific area of expertise, or prior collaborations with CDC. An ad hoc committee of CDC staff from several branches and programs within NCCDPHP observed the meeting to provide clarification on CDC programs and to comment on the recommendations.

The meeting was facilitated by representatives of the Institute for Alternative Futures (IAF), a nonprofit futurist organization that conducts futures research, visioning, and strategy development for nonprofits, associations, and governments. IAF was responsible for designing the interactive and creative meeting to lead the panel in developing a set of actionable recommendations.

Before the meeting, the panel reviewed supplemental materials, including a brief history of community health promotion activities at CDC, several examples of socioecological models for health promotion, and a summary of CDC-sponsored cutting-edge initiatives. During the first day of the meeting, participants discussed socioecological models and CDC's various roles in community health promotion (e.g., leader/advocate, standards developer, knowledge disseminator/translator). On the second day, the panel separated into subgroups to discuss innovative initiatives and to propose and refine recommendations for furthering community health promotion efforts. The larger group then reconvened to review and categorize the recommendations. Later, IAF summarized the notes of the meeting and consolidated all of the panel's proposals into eight specific recommendations. The CDC ad hoc committee met to review and prioritize the recommendations according to their appropriateness and feasibility.

In June 2006, the panel held a conference call to review a draft report of the discussions and the final recommendations proposed at the March meeting. Members considered the following issues: 1) Were the recommendations a strategic advance in community health promotion for CDC? 2) Do they align CDC with other leaders in community health promotion? 3) Was the report faithful to the intent of the recommendations proposed by the panel?

The final report was revised and disseminated as a white paper to the panel, the CDC ad hoc committee, CDC division directors, and other audiences.

## Recommendations

Discussions of socioecological models and CDC roles served as a creative platform to stimulate discussion and guide the formation of the recommendations. The panel acknowledged that ecological models have been useful in furthering community health promotion efforts, particularly as they relate to environmental influences on health status and behavior. In addition, the panel acknowledged CDC's valuable role in validating effective practices of community health promotion and supporting community-based participatory research and public health surveillance. The panel also affirmed CDC's role in disseminating public health knowledge across communities and serving as the voice among federal agencies for community health promotion.

However, the panel noted critical gaps in current ecological approaches and in CDC's current approach to health promotion. For example, even the best operational measures of the socioecological approach missed critical opportunities for change, including mental health and wellness, spirituality, and complementary and alternative medicine; access to care; political and economic contexts of decisions; race, racism, and discrimination; cultural beliefs and values as risk factors and protective factors; and elements of community efficacy, such as social capital and community competencies. The panel reported the need for an ecological approach to be sufficiently flexible to allow community choices based on available resources and local realities. Additionally, future approaches should facilitate discussions on power relationships, the political process, chronic social stressors (e.g., poverty), acute situations (e.g., hurricanes), and the engagement of nontraditional partners. Furthermore, the panel suggested that NCCDPHP extend its leadership role by looking beyond the traditional view of health to investigate what truly constitutes wellness in our society in partnership with universities, state and local health agencies, private organizations, nonprofit community institutions, and communities across the country.

The expert panel offered eight recommendations for NCCDPHP over the next 3 to 5 years related to community-based research and surveillance, training and capacity building, new program directions, and federal investments.

### A New Emphasis for Research and Surveillance

1. **Enhance surveillance systems beyond the tracking of individual risk factors to include community health indicators and social determinants of health.** A large and growing body of literature documents correlations between education, employment and income status, housing quality, and neighborhood safety and their impact on health outcomes. CDC is noted as a world leader in supporting surveillance systems that are critical to monitoring health trends, health status, and emerging health concerns. However, the agency does not routinely collect and report community health and social indicator data. Adding surveillance of social indicators will facilitate the development, implementation, and evaluation of health policies and public health interventions intended to reduce the burden of chronic diseases.

2. **Promote community-based participatory research (CBPR) within and outside of CDC.** CBPR is a collaborative, ecological approach to research that promotes the balance of knowledge acquisition with social action and equitably involves all partners, recognizing the strengths that each brings (15). Specifically, CBPR engages community members and researchers in setting and implementing a collaborative research agenda, thereby enhancing the relevance of research and increasing the likelihood of sustained and lasting change. Although CDC programs such as the Prevention Research Centers are engaged in CBPR, NCCDPHP could further promote this approach by 1) providing long-term investments in CBPR to research and evaluate social determinants of health and health disparities; 2) reenergizing the Federal Interagency Working Group on CBPR and supporting joint calls for proposals; 3) developing tools for community coalitions to produce reliable research findings and interventions; and 4) disseminating CBPR methods across CDC and to health departments, local communities, and organizations.

### Building Capacity for Community Health Promotion 

3. **Support training and capacity building to ensure that the public health workforce has the knowledge, skills, and tools necessary to implement community health promotion approaches.** The existing public health workforce is not fully equipped to implement a socioecological approach to disease prevention and health promotion. Working within a socioecological framework will require new practice, knowledge, and skills, as well as a shift in how the public health workforce currently conceptualizes and administers public health interventions. Competencies for training and capacity building to implement a socioecological approach would include cultural competence, advocacy, policy development, evaluation, use of community indicators, development of partnerships, and use of new communication technologies.

4. **Promote a state-of-the-art e-mechanism to share expertise and knowledge about community health promotion.** A more sophisticated electronic venue with the most recent and cutting-edge information about community health improvement is needed for the interactive exchange of information between communities and practitioners. The Web offers a platform for creating a virtual center for this expertise, which would facilitate the sharing of knowledge, evidence-based programs, and promising practices, and promote dialogue between communities, CDC, and other community health experts. Key elements of this Web-based platform would include data, communication tools, evidence-based research, training, and a forum for communities to contribute and share knowledge.

### Investing in New Approaches for Health and Wellness

5. **Champion a focus on wellness that includes mental health, spirituality, and complementary and alternative medicine (CAM).** Fragmentation and overemphasis on the physical aspects of health exclude mental health, spirituality, and CAM as integral parts of wellness. Although bringing these approaches together is complex, incorporating these practices into existing CDC programs and new initiatives with the input of diverse community voices would be an important step in furthering disease prevention and health promotion efforts. Critical steps include funding research on the influence of mental health, spirituality, and CAM practices on community health outcomes; assessing the influence of culture on mental health, spirituality, and CAM practices; building relationships to encourage parity in mental health care, such as reimbursement for mental health therapies; and addressing gaps in mental health services for specific population groups, including older adults.

6. **Shift a measurable part of NCCDPHP's community health promotion programs to focus on improving living conditions across the lifespan.** Ultimately, improving the public's health means intervening at both the individual and environmental level. The places (communities) in which people work and live have a large impact on health. Factors that affect living conditions include psychosocial factors, socioeconomic status, and natural (e.g., air, water) and built environments (e.g., transportation, water and sanitation, housing, buildings, green spaces, roads, other dimensions of urban planning) (10). In particular, negative living conditions such as toxic buildings and lack of green spaces disproportionately affect those who are already at an economic and social disadvantage. Moreover, it is also important to recognize developmental and historical processes over time at both societal (e.g., demographic changes) and individual levels (e.g., life course issues) (10). The panel recommended applying resources to interventions that focus on 1) changing living conditions strongly associated with health; 2) implementing evidence-based and practice-based interventions; 3) conducting evaluation and surveillance; and 4) promoting culturally tailored interventions by incorporating cultural competency and health literacy in policy and environmental interventions to address health disparities.

### Instituting Changes in Federal Investments

7. **Maximize the impact of federal resources dedicated to community health promotion.** The panel clearly articulated the perspective that federal resources can be maximally effective only through collaboration at all levels. Facilitating greater collaboration and coordination across federal agencies would include exploring programs and pilot projects of sister federal agencies to assess funding streams and points of collaboration; pooling funds and expertise and creating cross-cutting, collaborative, and integrative programs in health promotion and prevention of chronic disease; and seeking input from local communities to guide federal planning and decision making related to funding opportunities. Furthermore, federal regulations on spending must be modified to expedite and support such cross-agency collaborations.

8. **Provide funding tailored to the realities of community health.** Enormous investments of time, technical assistance, and resources are needed to build successful community programs. However, burdens on programs hinder the establishment of healthy communities. Such burdens include limited funding, short time frames to demonstrate impact, and restrictions on flexibility, which is critical for addressing changing circumstances. NCCDPHP could maintain and improve successful community programs by using integrated, long-term funding that is sufficiently flexible to meet the unique needs of local communities. Necessary actions would include providing community projects with appropriate guidance to ensure that evidence-based interventions are implemented; maintaining and enhancing funding for exemplary community programs such as REACH 2010 and STEPS to a HealthierUS; and encouraging communities to conduct a continuous quality-improvement process so programs can be modified based on the local context and changing needs.

## Conclusion

The leading causes of death and disability in the United States — heart disease, cancer, and stroke — are among the chronic diseases that have well-known behavioral, social, and environmental risk factors (16). Through NCCDPHP, CDC has pioneered and sustained a rich legacy of community-based health promotion and chronic disease prevention programs that have contributed to reversing trends in chronic disease mortality and morbidity. Working in concert with a range of governmental and private partners, CDC has developed a strong portfolio of accomplishments in the area of individual behavior change and a growing slate of health policy and environmental change interventions that are driving efforts across the nation to support healthy people and healthy communities.

Convening the National Expert Panel on Community Health Promotion provided the opportunity to conduct a critical external review of existing community health programs supported by NCCDPHP and to solicit the input of our nation's leading health promotion scholars, researchers, and practitioners in defining advances in community health promotion that can be championed by CDC. The recommendations put forth by the expert panel map a comprehensive socioecological approach to community health promotion that ranges from building surveillance systems that monitor social determinants of health to developing public health programs that promote mental health and wellness throughout the life stages. CDC, in collaboration with its partners and sister federal agencies, is uniquely positioned to provide significant scientific and programmatic leadership for evidence-based interventions that build healthy communities and eliminate health disparities. We view the work of the expert panel as a national call to action to multiple sectors of the public health system and a strategic advance for CDC.
